# Abemaciclib Therapy Using the MonarchE Criteria Results in Large Numbers of Excess Axillary Node Clearances—Time to Pause and Reflect?

**DOI:** 10.3390/cancers16173072

**Published:** 2024-09-04

**Authors:** Daniel Ahari, Mark Wilkinson, Nisha Ali, Vicky P. Taxiarchi, Rajiv V. Dave, Ashu Gandhi

**Affiliations:** 1 Wythenshawe Hospital and Nightingale Breast Cancer Centre, Manchester University Foundation Trust, Manchester M23 9LT, UK; daniel.ahari@mft.nhs.uk (D.A.);; 2 Centre for Women’s Mental Health, Division of Psychology and Mental Health, Faculty of Biology, Medicine and Health, University of Manchester, Manchester M13 9PL, UK; 3 Division of Cancer Sciences, Faculty of Biology, Medicine & Health, University of Manchester, Manchester Academic Health Sciences Centre, Manchester University Foundation Trust, Manchester M13 9PL, UK

**Keywords:** abemaciclib, axillary node clearance, monarchE study, CDK4/6 inhibitors

## Abstract

**Simple Summary:**

Abemaciclib is an important addition to the care of women with hormone receptor-positive breast cancer. To qualify for abemaciclib treatment, some women are advised to undergo axillary node clearance surgery as finding more than three axillary nodes with metastatic cancer allows access to abemaciclib. This paper explores the balance between the benefits of axillary node clearance in permitting access to abemaciclib and the harms of surgery. We examine how many women need to undergo axillary node clearance before one woman clinically benefits from the procedure. We show that for every 10 women undergoing axillary node clearance surgery, only one eventually qualifies for abemaciclib. The remaining nine would have axillary surgery but still not qualify for abemaciclib as less than four metastatic axillary nodes are found despite full axillary clearance. However, these women could still suffer the complications of axillary node clearance surgery.

**Abstract:**

The monarchE study added the CDK4/6 inhibitor abemaciclib to the care of women with oestrogen-positive (ER+) breast cancers. Eligibility required meeting monarchE criteria—either >3 positive axillary nodes, or 1–3 positive sentinel nodes (SNB+) with tumour size >50 mm or grade 3 cancers. Women were advised to proceed to completion axillary node clearance (cANC) if size/grade criteria were not fulfilled for >3 positive nodes to be identified. However, cANC is associated with significant morbidity, conflicting with the potential benefits of abemaciclib. We analysed data of 229 consecutive women (2016-2022) with ER+ breast cancer and SNB+ who proceeded to cANC, keeping with contemporary treatment guidelines. We used this cohort to assess numbers that, under national guidance in place currently, would be advised to undergo cANC solely to check eligibility for abemaciclib treatment. Using monarchE criteria, 90 women (39%) would have accessed abemaciclib based on SNB+ and size/grade, without cANC. In total, 139 women would have been advised to proceed to cANC to check eligibility, with only 15/139 (11%) having >3 positive nodes after sentinel node biopsy and cANC. The remaining 124 (89%) would have undergone cANC but remained ineligible for abemaciclib. Size, age, grade, and Ki67 did not predict >3 nodes at cANC. Following cANC, a large majority of women with ER+, <50 mm, and grade 1–2 tumours remain ineligible for abemaciclib yet are subject to significant morbidity including lifelong lymphoedema risk. The monarchE authors state that 15 women need abemaciclib therapy for 1 to clinically benefit. Thus, in our cohort, 139 women undergoing cANC would lead to one woman benefitting.

## 1. Introduction

Oestrogen receptor-positive (ER+), human epidermal growth factor receptor 2 negative (HER2−) breast cancers comprise the majority of breast cancer diagnoses and, as a proportion of all breast cancers, are increasing in incidence [1]. Traditionally, women with ER+ breast cancers have been treated with hormone blocking agents such as tamoxifen and aromatase inhibitors [2,3], with or without ovarian function suppression in premenopausal women, with significantly improved outcomes. There is a demonstrated survival benefit following adjuvant chemotherapy for women with ER+ breast cancers [4], the utility of which has been further refined by the development of tumour gene expression profiling tests. However, despite maximal treatment, long-term outcomes can remain poor with distant recurrence rates as high as 41% depending on tumour size and nodal status at diagnosis [5].

Newer treatments attempting to improve outcomes for women with ER+ breast cancer have targeted the cyclin-dependent kinase 4 and 6 (CDK4/6) protein pathways within breast cancer cells. By inhibiting intracellular CDK4/6 proteins, tumour cell cycle progression and cell division is blocked with potential therapeutic benefit [6]. CDK4/6 inhibitors are administered in conjunction with anti-oestrogenic agents, and of three such compounds available in breast cancer care, abemaciclib is the agent thus far shown to provide the most potential clinical benefit [7].

Abemaciclib’s efficacy in treating patients with ER+ breast cancer was investigated in the monarchE trial [7]. The inclusion criteria were patients with ER+ HER2− breast cancer with four or more positive axillary lymph nodes, or one to three positive nodes with either a tumour 5 cm or larger or grade 3 disease. Such patients comprised ‘cohort 1′ (91% of the 5637 patients), and a further ‘cohort 2′ (9%) investigated patients with the same receptor profile with 1–3 positive nodes and the additional feature of Ki67 of 20% or more, without the tumour size and grade criteria. Analysis at four years demonstrated an increased invasive disease-free and distant relapse-free survival (6.4% and 5.9%, respectively) compared to endocrine therapy alone, though no improvement in overall survival was seen [7].

As a result of the monarchE trial, abemaciclib’s use in women with higher-risk ER+ HER2− breast cancer has now become part of national guidance [8]. To establish eligibility for abemaciclib, the monarchE criteria are followed: in the scenario where patients with ER+ breast cancer have a tumour size of less than 5 cm or a histological grade of 1 or 2, the finding of macrometastatic sentinel nodes might result in advice towards completion axillary node clearance (cANC), also known as axillary lymph node dissection or ALND [9], to achieve the eligibility of four macrometastatic nodes. The intent of cANC in these women is to surgically remove all axillary nodes and send for histological analysis, in order to establish how many further nodes, if any, contain macrometastatic deposits over and above those seen in the sentinel node biopsy (SNB) [9].

Axillary node clearance may cause complications that can be significant for some women. The ALMANAC trial reported the presence of the following symptoms at 18 months post-operatively: pain (8.6%), numbness (19.0%), and reduced range of motion (8.4%) [10]. Surgical damage from the cANC to the lymphatic drainage system of the ipsilateral arm may lead to lymphoedema, the accumulation of lymph in the soft tissues [11]. Symptoms and signs in the affected limb may include a feeling of heaviness, substantial swelling, increased predisposition to infection, and pain [11]. The incidence of lymphoedema can be up to four times higher in patients who have undergone axillary node clearance ANC versus solely SNB [12].

Recognising the clinical tension between the potential benefit of abemaciclib if cANC demonstrates additional axillary node macrometastases versus the potential morbidity as a direct result of cANC we examined the outcomes of a historic cohort of women with ER+ HER2− breast cancer who had at least one macrometastatic sentinel node. Based on local and national treatment guidance in place at that time (2016–2022) [13], all of these patients proceeded to cANC. We applied current guidelines for assessing eligibility for abemaciclib therapy [8] to this historic cohort of women to analyse the numbers of women that would have potentially fulfilled the monarchE criteria on breast surgery and SNB results alone and the numbers that, according to *current* national guidance, would be advised to proceed to cANC to check eligibility for abemaciclib treatment. This would inform how many women had further axillary node macrometastases and would have actually proceeded to abemaciclib treatment and how many would not, i.e., would not have received any clinical benefit from the cANC. By performing this analysis in a retrospective cohort of women, we gain information that may assist current and future clinical decision-making with patients when cANC is considered an option to explore potential eligibility for abemaciclib therapy.

## 2. Methods

We reviewed all consecutive patients meeting the inclusion criteria who were treated at a University breast cancer centre over the course of July 2016 to June 2022. This project was registered with the hospital trust’s audit department. No ethical approval was required as this was a retrospective collection and analysis of fully anonymised data Data usage adhered to General Data Protection Regulations (UK).

To be included, a patient satisfied all the inclusion criteria, as follows:

1. Invasive breast carcinoma, stages T1–T3;
2. Tumour receptor status confirmed as oestrogen receptor-positive, human epidermal growth factor-negative (ER+ HER2−);
3. Sentinel node biopsy as the first axillary procedure with at least one sentinel node containing macrometastases, thus leading to completion axillary node clearance as a secondary procedure.

Our exclusion criteria were as follows:

4. Previous ipsilateral breast carcinoma;
5. Inflammatory breast carcinoma;
6. Systemic neo-adjuvant therapy;
7. Stage T4 carcinomas;
8. Male patients.

A search was performed on the Telepath laboratory information management system to identify all patients with histopathology reports for a breast excision specimen, a sentinel node biopsy, and an axillary clearance specimen during the period from July 2016 to June 2022. During this six-year period, all women satisfying the inclusion criteria for this analysis would have had breast surgery (breast-conserving surgery or mastectomy) plus SNB and then proceeded to a cANC on the advice of the breast cancer multidisciplinary team if the SNB showed macrometastatic deposits within the sentinel node(s). This was in line with local and national protocols in place at that time.

For each of these patients, the pathology reports were reviewed against the inclusion and exclusion criteria to confirm compatibility.

Patient demographics, tumour size, tumour grade, tumour biomarker status, SNB result, and axillary node clearance result were recorded.

Current national guidance [8], based directly on monarchE trial criteria [7], states that women with ER+, HER2−, early breast cancer are eligible for abemaciclib therapy in the following cases:

(a) There are more than 3 axillary nodes with macrometastatic deposits;
(b) There are 1 to 3 axillary nodes with macrometastatic deposits, plus at least one of the following criteria:
  - Grade 3 disease;
  - Primary tumour size ≥ 50 mm.

We applied the above, current, treatment guidance to our historic cohort of women with grade 1–2 hormone receptor-positive early breast cancer and 1–3 sentinel lymph nodes with macrometastatic deposits to see how many, if being treated today, would be advised to proceed to cANC solely for the purposes of determining eligibility for abemaciclib. Tumours were considered ER+ and HER2− in accordance with the Royal College of Pathologists dataset pertaining to pathology reporting of breast disease [14].

### Statistical Methods

This study is reported according to the STROBE guidelines for observational studies [15]. We provide descriptive statistics of clinical characteristics according to eligibility for abemaciclib treatment after (i) initial breast surgery and SNB, or (ii) further assessment with cANC. We tabulated the covariates of interest: age, invasive tumour size at diagnosis (in mm), histological grade, and ki67. Continuous variables are presented by the mean (standard deviation [SD]) or median (Interquartile range [IQR]) as appropriate. Categorical variables are presented by frequency (percentage). The association between each covariate and eligibility for treatment was examined using t-tests or Mann–Whitney non-parametric tests for continuous variables, and χ^2^ or Fisher exact tests for categorical variables, as appropriate.

The number needed to treat (NNT), i.e., to undergo cANC for one patient to meet the eligibility for treatment criteria, was estimated with a 95% CI. Provided that the probability of finding patients to meet the criteria for treatment if they had not undergone ANC would be zero (0), the formula to estimate the NNT was the reciprocal of the probability of meeting the criteria (1/p).

Lastly, the independent predictors of treatment eligibility of those undergoing ANC were examined with a logistic regression model, adjusting for all covariates.

The analysis was implemented using the statistical software Stata MP, version 16.1.

## 3. Results

Of the 229 women included in this study, 90 (39.3%) would have fulfilled the criteria for abemaciclib therapy after initial breast surgery and SNB, and 139 (60.7%) women would not have fulfilled the criteria (Table 1).

These 90 women fulfilling monarchE criteria would, in accordance with current national guidance [8], not require a cANC to be considered eligible for abemaciclib. In this group, the mean age was 56.6 years (SD 12.8), the mean tumour size in millimetres (mm) was 29.0 (SD 11.7), and five women (5.6%) had a tumour size greater than or equal to 50 mm. The majority of them had grade 3 disease (n = 86, 95.6%), and the remaining four (4.4%) had grade 2 disease. No woman had grade 1 disease. Reflecting the high incidence of grade 3 cancers in this cohort, the median Ki67 was 30% (IQR 22%, 35%).

The remaining 139 women with macrometastatic deposits in 1–3 sentinel nodes but who did not fulfil the initial monarchE tumour size or grade criteria for abemaciclib treatment would, by current national guidance [8], be advised to undergo a cANC solely for the purpose of assessing eligibility for abemaciclib therapy. This group of 139 women had a mean age of 60.6 years (SD 12.0) and a mean tumour size of 23.2 mm (SD 9.1). There were 18 (13.0%) women with grade 1 disease, and the remaining 121 (87.0%) had grade 2 disease. The median Ki67% was 15 (IQR 10, 20). None of them had a tumour greater than or equal to 50 mm, nor did any have grade 3 disease.

Based on the results of their cANC, this group of 139 women can be further divided as follows:

(i). Those women in whom cANC resulted in an overall total (macrometastatic SNB plus macrometastatic nodes within the cANC) of less than four macrometastatic axillary nodes and would thus be ineligible for abemaciclib (n = 124, 89.2%);
(ii). Those women in whom cANC resulted in an overall total of four or more axillary nodes with macrometastases and thus would be eligible for abemaciclib (n = 15, 10.8%) (Table 1).

Of these 15 women who would qualify for abemaciclib after cANC, the mean age was 60.0 years (SD 13.4) and mean tumour size was 26.5 mm (SD 10.1). Regarding disease grade, two (13.3%) women had grade 1 disease and 13 (86.7%) had grade 2 disease. The median Ki67% was 16 (IQR 12, 25). Of the 125 women who would not qualify for abemaciclib after cANC, the mean age was 60.6 (SD 11.9) and the mean tumour size was 22.7 mm (SD 8.9). Regarding disease grade, 16 (12.9%) women had grade 1 disease and 108 (87.1%) had grade 2 disease. The median Ki67% was 14 (IQR 10, 20). In the group of 15, the mean total number of axillary nodes removed was 17.5 (range 9–30).

**Figure 1:**
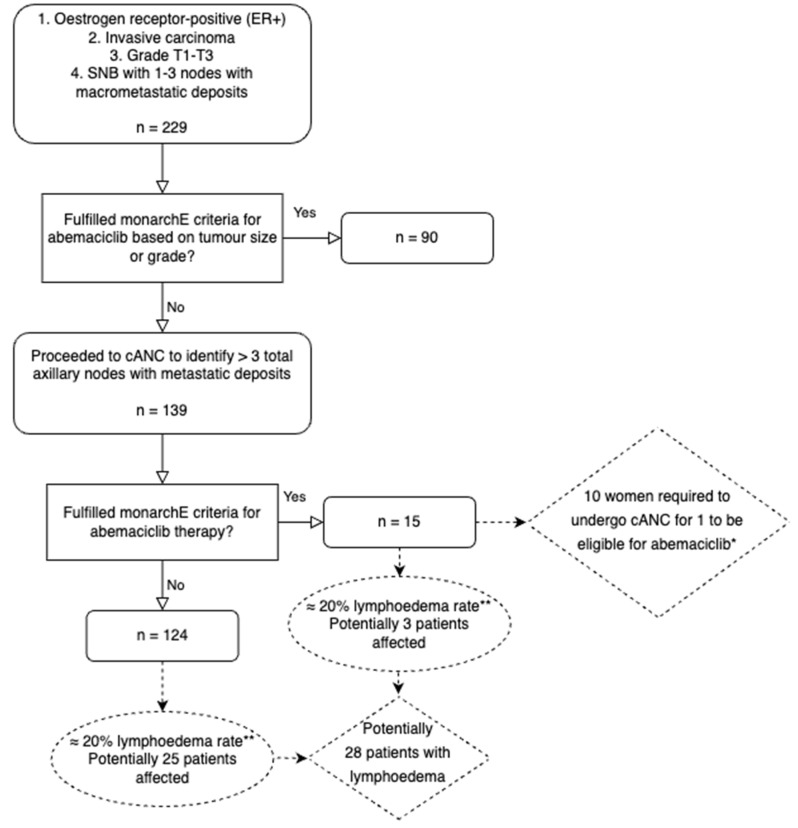
Consort diagram of 229 women with oestrogen receptor-positive (ER+) breast cancer diagnosed between 2016 and 2022 assessed for suitability for abemaciclib therapy using monarchE criteria [7]. SNB = sentinel node biopsy, cANC = completion axillary node clearance. * Data calculation described in Methods section. ** Lymphoedema rate derived from DiSipio et al. [12].

It therefore follows from the above that, in calculating the number needed to treat, it would require 10 (95% CI 6, 17) women to undergo cANC for 1 to be eligible for abemaciclib (Figure 1).

We found no indication that any covariate is an independent predictor of eligibility for abemaciclib at cANC (Table 2), nor did numbers of axillary nodes with macrometastases at initial SNB predict eligibility for abemaciclib following subsequent cANC (Supplementary Table S1).

## 4. Discussion

Given that one key component of eligibility for abemaciclib is nodal burden, in a woman whose tumour lacks size or grade criteria but who has macrometastatic sentinel nodes, the decision to recommend completion axillary node clearance may appear attractive, given that it allows histological assessment of all nodes for disease involvement and thus facilitates access to extra systemic treatment (abemaciclib) that would have otherwise been denied. Clinical conflict then arises between the potential benefit of abemaciclib if enough axillary nodes have macrometastases versus the potential harm of lymphoedema as a direct result of cANC.

As abemaciclib therapy is now recommended as national guidance [8], the balance between the benefits and harms of cANC again warrants further discussion.

Using a large cohort of women presenting to a University Hospital Breast unit with ER+ HER2− breast cancer who all proceeded to cANC following initial surgery showing SNB macrometastases, we conducted this study to examine, using the monarchE criteria [7], the proportion of women who, as a result of undergoing cANC that yielded a total of four or more axillary lymph nodes with macrometastases, would be able to proceed to abemaciclib treatment.

In our cohort of 229 women, 90 fulfilled the criteria for abemaciclib from the results of the initial breast surgery and SNB alone, without the need to proceed to cANC (though, in this retrospective series, they did eventually have cANC as this was the local and national guidance in place at that time). The remaining 139 (61%) would not have fulfilled the criteria for abemaciclib from initial breast surgery and SNB alone and, in current practise, would have been advised to progress to cANC to establish potential eligibility for abemaciclib therapy. Following cANC, 15 (11%) patients would have fulfilled criteria due to the cANC proving further axillary node involvement.

The remaining 124 (89%) patients saw no discernible clinical benefit from undergoing cANC but would be exposed to the physical complications of this procedure. Compared to breast cancer survivors without lymphoedema, those affected by lymphoedema have worse health-related quality-of-life scores, with poorer body confidence, depression, and anxiety [11,16]. In addition to the consequential mental and physical costs to patients who develop lymphoedema, recent randomised controlled trials and systematic reviews have also demonstrated the significant financial burden imposed on patients and healthcare systems [17,18].

For all women proceeding to abemaciclib therapy, the monarchE authors report that the number needed to treat to see 1 woman benefit clinically is 15 [19]. Thus, in our cohort, of the 139 women undergoing cANC, applying the monarchE criteria and the monarchE authors’ own numbers we can conclude that 1 of these 139 women (0.7%) would have gained clinical benefit from undergoing ANC if current guidelines for abemaciclib therapy were in place at the time of their breast cancer care. Lymphoedema rates are known to be approximately 20–44% following ANC [12,20]; therefore, in this cohort of 139 women, potentially as many as 28 women could be suffering with lymphoedema.

If women with ER+ breast cancer and a positive SNB are offered the option to undergo cANC to assess eligibility for abemaciclib (in the cases where tumour size and/or grade does not suffice for this purpose) then, in addition to the information that, currently, there is no improvement in overall survival yet demonstrated following abemaciclib therapy [7], they should be fully informed of the equipoise between the harm and benefit of cANC in their particular situation. Furthermore, the use of surrogate end points such as progression free survival (used in the monarchE study) has been shown to have poor correlation with overall survival [21]. Our data suggest that, in those women where cANC is needed to demonstrate eligibility for abemaciclib, almost 9 out of 10 of these women will undergo cANC for no clinical benefit. Approximately 1 in 10 will qualify for abemaciclib therapy as a result of their cANC (but the monarchE authors report that of 15 patients qualifying for abemaciclib therapy, 1 patient will derive clinical benefit). In addition to other possible complications of ANC, potentially 2 out of every 10 patients will suffer lifelong lymphoedema.

Tinterri et al. have recontextualised the findings of the SINODAR-0NE randomized clinical trial in light of monarchE’s findings [22,23]. The study showed that in patients undergoing breast-conservation surgery or mastectomy, omission of ANC does not lead to worse survival or recurrence rates and that whilst a minority of breast cancers may be understaged by omitting ANC, other treatment options sufficiently mitigate this. They do, however, specifically advocate for cANC where three out of four of the above predictive factors are present.

Our data contribute to similar data emerging from other institutions. Williams et al. examined a cohort of women with ER+ HER2− breast cancer analogous to those in our study, finding that only 13% would derive candidacy for abemaciclib as a result of >4 macrometastatic axillary nodes being identified with cANC [24]. Similarly, Gaillard et al. found that, in their cohort of comparable patients, 12% would meet the required axillary nodal criteria for abemaciclib therapy following cANC for this purpose [25]. Our data build on these two aforementioned studies in that they present an analysis of a contemporaneous cohort of women rather than those diagnosed up to 23 years ago [25], an important point given the rapid advances in breast cancer management. Furthermore, all women in our cohort followed a consistent treatment pathway with clarity on sequencing and type of surgery. We did not include women having neo-adjuvant systemic treatment and a variety of surgical approaches to the axilla as in other studies [24]. A limitation of our study is that the dataset is relatively smaller than the two contemporary studies. However, the completeness of the data allows us to present numbers needed to treat, i.e., the number of women required to undergo cANC for one woman to become eligible, on axillary nodal criteria alone, for abemaciclib—a useful concept when discussing treatment options with women and within multidisciplinary teams.

Other key limitations include the prospect of survivorship bias: only patients who had both SNB and subsequent cANC were included—these patients may not be representative of all patients who have had an SNB and would have been eligible for a cANC but opted out. This study was also not randomised but was a retrospective analysis. Finally, it is noteworthy that the original monarchE study included patients from 38 countries, while our cohort is from a single centre in North West England, in that results may not be as generalisable.

## 5. Conclusions

As shown by our data and others [24,26], in the absence of other tumour biology criteria permitting abemaciclib therapy, performing cANC for the sole purpose of determining eligibility for abemaciclib results in large proportions of women receiving surgery that is of no clinical benefit yet imparts the possibility of lifelong arm morbidity. We subscribe to the concept that the absence of cANC will possibly lead to understaging in a minority of women, as it will very rarely lead to under-treatment [27].

## Figures and Tables

**Table 1:** Description of patients who met criteria [7,8] for abemaciclib (A) after initial breast surgery and SNB, (B) following cANC.

(A)			
	Following initial breast surgery and SNB (n = 229)		
	Yes	No	*p*-value
N (%)	90 (39.3)	139 (60.7)	
Age, mean (sd)	56.6 (12.8)	60.6 (12.0)	0.018
Tumour size mm, mean (sd)	29.0 (11.7)	23.2 (9.1)	<0.001
Tumour size ≥ 50 mm (%)	5 (5.6)	0	0.009
Histological grade			<0.001
1	0	18 (13.0)	
2	4 (4.4)	121 (87.0)	
3	86 (95.6)	0	
Ki67, median % (IQR)	30 (22, 35)	15 (10, 20)	<0.001
(B)			
	Following cANC (n = 139)		
	Yes	No	*p*-value
N (%)	15 (10.8)	124 (89.2)	
Age, mean (sd)	60.0 (13.4)	60.6 (11.9)	0.845
Tumour size mm, mean (sd)	26.5 (10.1)	22.8 (8.9)	0.130
Tumour size ≥ 50 mm (%)	0	0	-
Histological grade			0.963
1	2 (13.3)	16 (12.9)	
2	13 (86.7)	108 (87.1)	
3	0	0	
Ki67, median % (IQR)	16 (12, 25)	14 (10, 20)	0.172

**Table 2:** No independent clinico-pathological predictors of meeting eligibility for abemaciclib in 139 women with grade 1–2, hormone-positive, HER2−negative, early breast cancer and <50 mm tumour diameter, undergoing cANC following SNB showing 1–3 nodes with metastatic deposits.

	OR (95% CI)	*p* Value
Age	1.00 (0.95, 1.04)	0.910
Invasive tumour size (mm)	1.05 (0.99, 1.12)	0.111
Histological grade		0.505
1	Ref.	
2	0.56 (0.10, 3.10)	
Ki67	1.04 (0.98, 1.10)	0.236

Full demographic data are available in Supplementary Table S2.

## Data Availability

The datasets generated during and/or analysed during the current study are not publicly available due to possible compromise of individual privacy but are available from the corresponding author on reasonable request.

## Supplementary Materials

The following supporting information can be downloaded at https://www.mdpi.com/article/10.3390/cancers16173072/s1, Table S1: Number of women whose additional axillary nodes were found to contain macrometastatic deposits following cANC; Table S2: Demographics.
