# Supplementary material for: Abemaciclib Therapy Using the MonarchE Criteria Results in Large Numbers of Excess Axillary Node Clearances—Time to Pause and Reflect?
Source: Cancers (Basel). 2024 Sep 4;16(17):3072. doi: 10.3390/cancers16173072 (PMC11394446; doi:10.3390/cancers16173072)
Supplement: Supplementary file 1 [file cancers-16-03072-s001.zip › cancers-3154867-supplementary.pdf]

Table S1. Numbers of women where additional axillary nodes were found to contain macrometastatic deposits following cANC.

Number of sentinel nodes with macrometastatic deposits at initial	Number of women undergoing cANC				
	0 additional nodes	1 additional node	2 additional nodes	3 or more additional nodes	
1	70	14	4	4	92
2	28	1	0	8	37
3	6	3	0	1	10
	104	18	4	13	139

Table S2. demographics.

	All patients (n =229)	Eligible for abemaciclib at SNB (n=90)	Ineligible for abemaciclib at SNB (n=139)		
			All (n=139)	Eligible for abemaciclib at cANC (n=15)	Ineligible for abemaciclib at cANC (n=124)
**Age**					
Mean	58	56	60	59	60
Range	25 – 87	25 – 82	29 – 87	43 - 80	29 – 87
Median	57	55	59	57	59
LQ - UQ	49 - 69	47 – 66	52 - 69	47 - 70	52 – 69
**ER+**	229 (100%)	90 (100%)	139 (100%)	15 (100%)	124 (100%)
**HER-**	229 (100%)	90 (100%)	139 (100%)	15 (100%)	124 (100%)
**Ki67%**					
Mean	22	31	16	18	16
Range	4 – 80	9 – 80	4 – 60	8 – 32	4 – 60
Median	20	30	15	16	14
LQ - UQ	12 – 30	22 – 35	10 – 20	14 – 23	10 – 20
**Size (mm)**					
Mean	25	29	23	27	23
Range	6 – 50	8 – 50	6 – 48	8 – 45	6 – 48
Median	24	28	22	27	22
LQ - UQ	17 - 33	21 – 39	17 – 28	22 – 33	16 – 28
**Grade**					
1	18 (7.9%)	0	18 (12.9%)	2 (13.3%)	16 (12.9%)
2	125 (54.6%)	4 (4.4%)	121 (87.1%)	13 (86.7%)	108 (87.1%)
3	86 (37.6%)	86 (95.6%)	0	0	0
